# Progress in Bacteriology

**Published:** 1903-07-11

**Authors:** 


### PROGRESS IN BACTERIOLOGY.

**Sleeping Sickness.**—The Portuguese Commission which investigated the etiology of this disease described as the causative agent a diplo-streptococcus which they found in the cerebro-spinal fluid, spleen and blood of negroes suffering from the complaint. This organism they proposed to call the hypnococcus. Castellani, of the English Commission, also described a streptococcus, but decided that this was an entirely different species of organism from that portrayed by Bettencourt[1] and his confreres. These Portuguese Commissioners, reverting again to the matter now claim that the two organisms are identical and that the supposed differences are qualitative only and trivial in character. It is differentiated from the pneumococcus by the fact that it does not produce an acid reaction in His's fluid. Growth on agar, gelatine, glycerine-agar and bouillon is seldom successful, and the best medium is that recommended by Martin, made by macerating the stomach of a pig. Any peptone used in media should be free from albumose. The hypnococcus does not coagulate milk, or form gas in glucose media. Isolation from the blood is a matter of difficulty, but the Portuguese Commissioners succeeded in two out of seven cases. There appears to be no doubt that the two organisms found associated with sleeping sickness in two widely separated parts of Africa, and by two totally independent groups of investigators, are in every way identical, and that the honour of priority of discovery lies with the Portuguese.

**Diphtheria.**—The June number of *Public Health* is devoted in large part to the consideration of diphtheria. Heaven[2] enforces the fact that the disease is one of person rather than place. He believes that epidemics may be checked by systematic bacteriological examinations of the throat and nose of suspected persons. Of especial interest is the importance which he attaches to nasal examinations, and the presence of short diphtheria bacilli. Whilst cases carrying typical bacilli must be isolated, those with short bacilli may be dealt with in their own homes or in the out-patient departments of hospitals. School closure is not as a rule advisable, as the inspection of the children is thus rendered more difficult. The short bacillus of the Hofmann type may on examination prove virulent; it may develop into the long typical variety; it may itself set up a fatal attack, so that the exclusion from school of children carrying this organism is in every way desirable. The charts and tables supplied with the article go to show that epidemic outbreaks can be controlled by systematic bacteriological weeding out. Martin[3] discusses the occurrence of diphtheria in animals and notes that the disease was first one of country life where the peasantry are brought into close contact with animals. Power first pointed out that possibly cows suffered from the disorder and so transmitted it through milk. Cats undoubtedly may be a source of infection, and virulent diphtheria bacilli have been found in the nasal discharge of a horse. Bacilli differing only very slightly from diphtheria bacilli have been found in the throats of diseased and healthy pigeons, and Salter claims that pseudo-diphtheria organisms may be exalted in virulence by passage through small birds. Diphtheric roup in fowls has been successfully combated by the use of diphtheria antitoxin. Martin suggests the possibility of diphtheria being communicated by the fleas and lice from fowls, dogs, and cats. Harrison,[4] however, denies that there is any connection between the diphtheria of fowls and that of human beings. He failed to infect fowls by injecting cultures of diphtheria bacilli, and found that diphtheria antitoxin had no protective or curative value in fowl's roup. The false membrane of roup has not the necrotic characters of the true diphtheria membrane, and was not pathogenic when inoculated upon rabbits and guinea-pigs. The organism associated with roup he styles the bacillus cacosmus, and roup may be produced in the hen not only by inoculation with this germ but also by the bacillus pyocyaneus and other organisms. Concetti [5] describes a variety of diphtheria bacillus which resembles the actinomyces, and which would place the organism in the streptothrix group. His description of this variety is as follows : "The bacilli are clavate in outline, not uniformly stained, but divided as it were into three or four fragments, with intermediate, unstained spaces. The contents of the bacilli were almost homogeneous, being but slightly granular." From such forms typical Klebs-Löffler bacilli were obtained. The clavate forms are apparently the result of continued cultivation, and have a very low toxicity, being almost parasitic. The author suggests that in the discovery of the life habits of these saprophytic forms which are widely distributed in nature must lie the future control of the disease. Forbes [6] found diphtheria bacilli in the ear discharges of scarlet fever patients in 32 out of 40 cultures. Long and medium-sized forms predominated, but in two cases in which the short forms were tested by inoculation they were found to be infective and virulent. The bacilli cannot generally be found until the second day of the attack of otorrhœa which is primarily caused by cocci. They are generally, however present in very large numbers so that they can be recognised in smear preparations, and they persist for many weeks or months. There is no distinctive sign of diphtheritic otitis, and antitoxin has neither prophylactic or curative effect. Infection of the ears takes place through the nose, and aural cases are fruitful sources of infection to others and should therefore be isolated. Denny [7] has studied the morphological differences between B. diphtheriæ, B. xerosis, and B. pseudo-diphtheria under various conditions of culture. In young cultures all alike are solid staining, paired, parallel rods, showing post-fission movements. B. pseudo-diphtheria never developes further than this. B. xerosis becomes an elongated rod with segmented protoplasm. B. diphtheriæ shows clubbed, barred, and filamentous forms, and occasionally true branching. The three varieties have all, probably, a common origin, and in the early stages of their development such as seen in four or eight hour cultures are indistinguishable from one another.

**Streptococci in Scarlet Fever.**—Weaver [8] summarises, as follows, his investigations into the vitality of streptococci from the throats of scarlet fever patients. Streptococci are constantly present in large numbers during the early stage, and more sparsely later in the disease. They resist drying as long or longer than other organisms present, and have been preserved in the dry state for as long as 90 days. They remain alive for a long time on milk, and grow best on any medium containing sugar. They cannot be distinguished by cultural or morphological peculiarities from other streptococci.

**Variola and Vaccination.**—Councilman, Magrath, and Brinckerhoff,[9] in a preliminary communication on the etiology of variola, describe an organism which is to be found in the lower epithelial cells of the skin. This organism is a small structureless body, from one to four microns in diameter. It undergoes complete cycles of development, first intracellular, and then intranuclear. The authors suggests that in small-pox the complete development of the parasite takes place through the two cycles, and that in vaccinia the primary cycle only is accomplished.

[1] Brit. Med. Jour., April 18. 1903. [2] Public Health, June, 1903. [3] Ibid. [4] Domin. Med. Menth., March, 1903. [5] Ann. d'Ig. Speriment., vol. xi., fasc. iij. [6] Jour. Pathol and Bact., May, 1903. [7] Ibid. [8] Jour. Med. Research, May, 1903. [9] Ibid.

